# Movable Kidney: Its Etiology, Pathology, Diagnosis, Symptoms and Treatment

**Published:** 1911-09

**Authors:** 


**Movable Kidney: its Etiology, Pathology, Diagnosis, Symptoms and Treatment.** By WILLIAM BILLINGTON, M.S., F.R.C.S. Pp. ix, 167. London: Cassell and Company Ltd. 1910.

The author has performed nephropexy upon three hundred and fifty kidneys, and the monograph before us is chiefly based upon the experience gained with these cases. The method of fixation used has been practically the same in all the cases, and is a composite one in which the suggestions of various surgeons have been utilised, and it appears to have been first employed by Mr. Jordan Lloyd. The technique includes partial decapsulation of the upper half of the kidney, and the employment of temporary supporting sutures which are attached to the capsule of the lower half of the kidney, and a special feature is the fixation of the kidney at the normal height, with the hilum on a level with the twelfth rib where it emerges from beneath the erector spinæ muscle. The author has no knowledge of any case of recurrence of mobility after the operation, nor of any subsequent operation being necessary.

It is remarkable that one surgeon should have operated so often for movable kidney in the space of a few years. In the absence of special contra-indications an attitude towards the operation seems to be taken up analogous to that taken by most surgeons with respect to the radical cure of hernia, with the difference that operation is not quite always recommended. It is recommended when the mobility of the kidney is held responsible for trouble of some kind or other, such as pain in various parts of the body, various disorders of the digestive, urinary or sexual organs, or of the nervous system, including neurasthenia, mental disorders and insanity.

We have found the book interesting and suggestive. Operative technique and the anatomy of the renal regions are lucidly described and well illustrated.

